# SGLT2 Inhibitors Correct Fluid Overload in Adult Kidney Transplant Recipients—A Prospective Observational Study

**DOI:** 10.3389/ti.2024.12879

**Published:** 2024-06-10

**Authors:** Anja Schork, Marie-Luise Eberbach, Ferruh Artunc, Bernhard N. Bohnert, Felix Eisinger, David J. Heister, Dorothea Vosseler, Silvio Nadalin, Andreas L. Birkenfeld, Nils Heyne, Martina Guthoff

**Affiliations:** ^1^ Department of Internal Medicine IV, Division of Diabetology, Endocrinology, Nephrology, University Hospital Tübingen, Tübingen, Germany; ^2^ Institute of Diabetes Research and Metabolic Diseases (IDM) of the Helmholtz Center Munich at the University of Tübingen, Tübingen, Germany; ^3^ German Center for Diabetes Research (DZD), Tübingen, Germany; ^4^ Department of Cardiology and Nephrology, Sindelfingen Hospital, Sindelfingen, Germany; ^5^ Department of General and Transplant Surgery, University Hospital Tübingen, Tübingen, Germany

**Keywords:** SGLT2 inhibitor, bioimpedance spectroscopy, kidney transplantation, glucosuria, fluid overload

## Abstract

In this longitudinal observational study, we measured urinary glucose concentration, body composition and volume status (bioimpedance spectroscopy) and plasma renin and aldosterone concentrations in *n* = 22 kidney transplant recipients (KTRs) initiating on SGLT2I at baseline (BL), and after 1 week and 1, 3, and 6 months. Estimated glomerular filtration rate (eGFR) decreased by −2 mL/min/1.73 m^2^ (IQR −10–0) after 1 week and remained stable thereafter. Urinary glucose concentration was 10 (3–24) g/g creatinine after 1 week and correlated with eGFR (r^2^ = 0.273; *p* = 0.057). SGLT2I did not affect HbA1c, fasting blood glucose, body weight, fat or lean mass. SGLT2I decreased fluid overload dependent on baseline overhydration (OH, r^2^ = 0.54, *p* = 0.0003) without occurrence of dehydration. Plasma aldosterone increased at day 7, while plasma renin did not change significantly. In conclusion, SGLT2I corrected fluid overload in patients with elevated overhydration at baseline, while in euvolemic KTRs fluid status remained stable without reduction of body water below the reference range, thus promoting the safety of SGLT2I therapy in patients following kidney transplantation. Glucosuria, together with effects of SGLT2I on blood glucose control and body weight, is attenuated in KTRs dependent on eGFR.

## Graphical Abstract

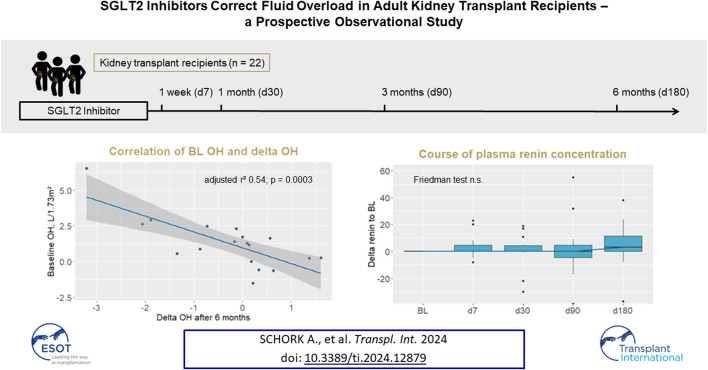

## Introduction

Comprehensive care of kidney transplant recipients (KTRs) aims to maximize kidney allograft survival and on top of that seeks to reduce the patients’ cardiovascular risk while balancing side effects of immunosuppressive therapy, including control of blood glucose, body weight, blood pressure, and fluid status [1]. SGLT2 inhibitors (SGLT2I) have emerged as an effective therapy to reduce proteinuria and progression in patients with chronic kidney disease (CKD) [2, 3]. Owing to their mechanism of action, SGLT2I modulate body weight, body composition and fluid status. In obese patients with type 2 diabetes mellitus and normal kidney function, we have used bioimpedance spectroscopy to investigate changes in body composition underlying the reduction of body weight with SGLT2I and observed a persistent reduction of adipose mass and a transient reduction of extracellular water after a few days [4]. This initial reduction of extracellular fluid was counter regulated by an increase of plasma renin activity and serum aldosterone concentration, and fluid status returned to initial values after 1–3 months [4]. Other studies also found a reduction of adipose mass after initiation of SGLT2I in diabetes patients, and some studies reported a reduction of lean tissue or muscle mass [5]. Latent or apparent fluid overload is frequently present in CKD, and is associated with disease progression, increase in systolic blood pressure and natriuretic peptides as parameters of cardiovascular stress [6]. Bioimpedance spectroscopy measurements of fluid status in CKD patients revealed a reduction of fluid overload by SGLT2I without occurrence of exsiccosis, again accompanied by activation of renin-angiotensin-aldosterone-system (RAAS) [7, 8].

Despite the numerous cardio- and reno-protective effects of SGLT2I, the use of SGLT2I in the vulnerable cohort of KTRs has not yet been studied in sufficient depth and is therefore still restrictive [9, 10]. Potential concerns in this cohort include the risk of acute kidney injury due to potential interference in volume homeostasis, and urinary tract infections. Use of SGLT2I in KTRs was initially described in several observational studies with encouraging results regarding safety and glycemic control [11–18]. In a small, randomized controlled trial with *n* = 49 patients, empagliflozin was safe, and improved glycemic control in stable KTRs with median eGFR of 66 (range 41–83) mL/min/1.73 m^2^ [19]. In a multicenter cohort study from South Korea, SGLT2I were shown to have beneficial effects on graft function in diabetic KTRs [20]. Although the study design was retrospective, this study was the first to demonstrate reno-protective effects in KTRs. More recently, a large observational study confirmed the benefits of SGLT2I treatment in *n* = 339 KTRs with regard to blood glucose control and reduction of proteinuria, and named urinary tract infections as the most frequent adverse event [21]. While this evidence is indicative of reno-protective effects of SGLT2I in KTRs, data on effects of SGLT2I on volume homeostasis after kidney transplantation is sparse. This study therefore investigated the effects of SGLT2I on urinary glucose excretion, body composition and fluid status in KTRs.

## Patients and Methods

### Study Design

Adult kidney transplant recipients from the transplant center outpatient clinic of the University Hospital of Tuebingen who had decided to be treated with SGLT2I between March 2021 and July 2022 were requested to participate in this longitudinal observational study. The decision to embark on an SGLT2I was made solely by the attending transplant nephrologist. Patients were included independently of CKD etiology, the presence or absence of diabetes, and the SGLT2I prescribed. Time since transplantation was at least 6 months. Specified time points for the study visits were baseline (BL, the day of the prescription of the SGLT2I) and 1 week, 1 month, 3 months, and 6 months of follow up (FU) after initiation of the SGLTI.

The study was approved by the local ethics committee of the University of Tuebingen (648/2016BO1). A written informed consent was obtained from all patients. The study was registered at the German Clinical Trials Register (DRKS00028560).

### Assessment of Body Composition and Fluid Status

At each study appointment, body composition and fluid status were measured using the Body Composition Monitor (BCM, Fresenius Medical Care). This device uses bioimpedance spectroscopy to detect fluid overload, and was initially developed to help to determine dry weight in patients undergoing dialysis [22]. The BCM differentiates between intra- and extracellular water by measuring bioimpedance with 50 frequencies between 5 and 1,000 kHz, whereby the low frequencies cannot pass cell membranes [23]. The BCM device calculates intracellular water (ICW) as the difference between extracellular (ECW) and total body water (TBW) on the basis of the “body volume model,” and parameters of lean and adipose tissue on the basis of the “body composition model.” Excess fluid, which is mainly located in the extracellular compartment, is calculated by the BCM device from normally hydrated lean and adipose tissue masses, and is defined as overhydration (OH). Reference values for OH in healthy individuals lie between −1 and +1 L [24]. Values obtained for OH, ECW, ICW, and TBW were normalized to a body surface area of 1.73 m^2^.

### Laboratory Measurements and Assessment of Urogenital Infections

Laboratory values were determined in the central laboratory (Institute for Clinical Chemistry and Pathobiochemistry) of the University Hospital of Tuebingen. In addition to the standard of care diagnostic parameters after kidney transplantation, plasma renin and aldosterone concentrations were measured in plasma samples and urinary glucose concentration was measured in a spot urine sample. The patients were monitored for urogenital infections by medical history and urinary dipstick.

### Statistical Analysis

Parameters are reported with number and percentage (nominal parameters) or median and quartiles (continuous parameters) and illustrated as absolute values or delta values from baseline. Friedman test was performed to test for changes during the complete follow up. Wilcoxon-Mann-Whitney-Test (Wilcoxon signed rank test, referred to in short as Wilcoxon test) for paired samples was performed to test for differences between two respective follow up time points. Bonferroni correction was used to correct for multiple testing. A linear regression model was fitted for univariate correlations.

Statistical significance was defined as a significance threshold of *p* < 0.05. The statistical software packages R studio version 4.1.2 and Microsoft Office Professional Plus 2019 Excel version 1808 were used to perform data analysis.

## Results

### Characterization of the Study Cohort

**FIGURE 1:**
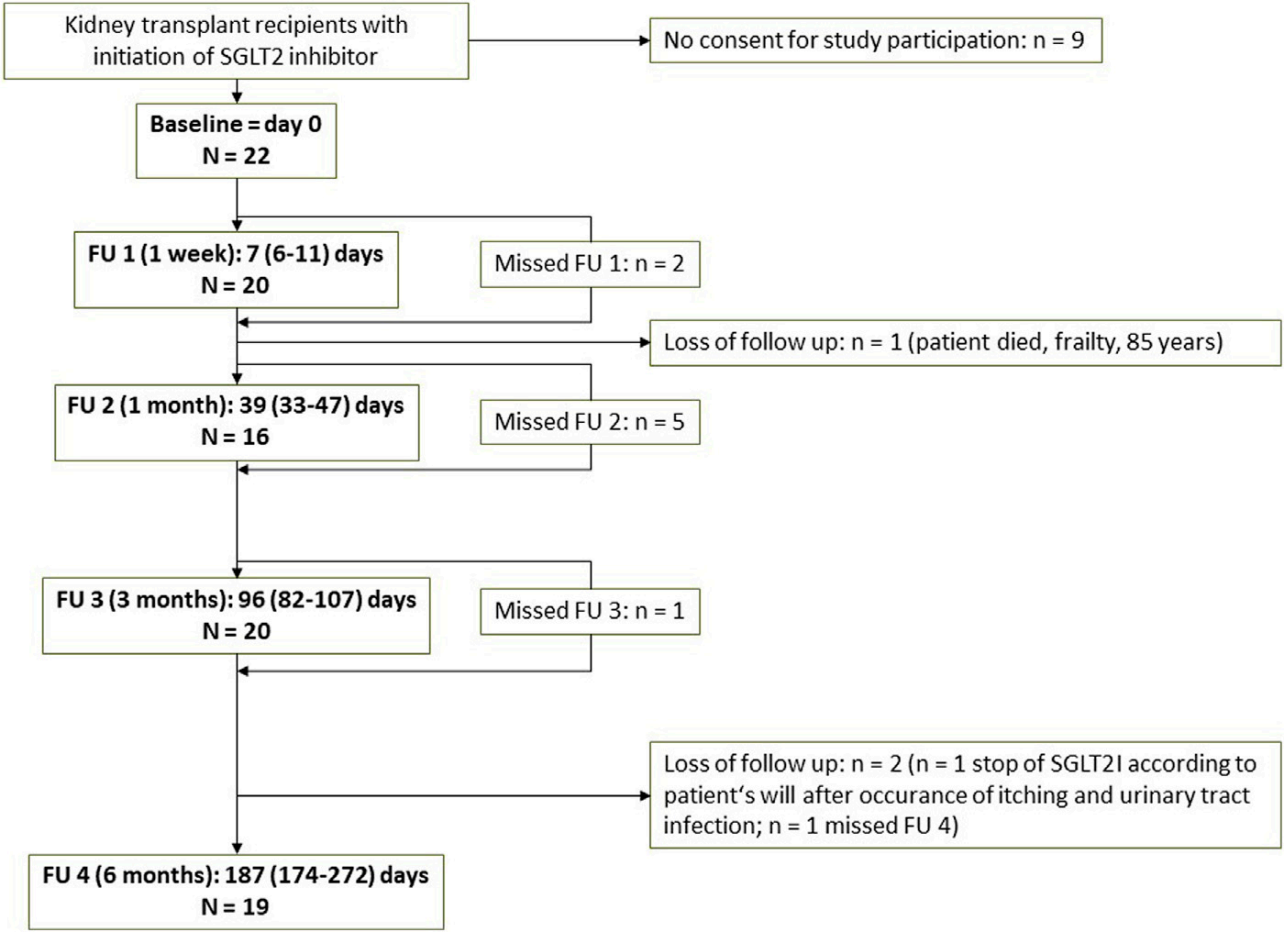
Flowchart of the study Abbreviations: FU, follow up; SGLT2I, SGLT2 inhibitor.

A total of 22 patients were included in the study, and follow up after 6 months was available in *n* = 19. Actual time points of all FU visits and number of patients are shown in the flowchart (Figure 1). N = 1 patient died during follow up due to age (85 years) and frailty. N = 1 patient terminated dapagliflozin due to an itchy rash on the forehead (temporal relation with the initiation of dapagliflozin, however causal relation uncertain, itching was not completely resolved after termination of dapagliflozin) and urinary tract infection. Other missing values are due to missed study visit of a patient due to the long distance to the transplant outpatient clinic.

Baseline patient characteristics are displayed in Table 1. Diabetes mellitus was present in *n* = 18 study patients (*n* = 10 with pre-existing diabetes and *n* = 8 with post-transplantation diabetes mellitus, PTDM) and *n* = 2 patients had elevated HbA1c without manifest diabetes mellitus. Blood glucose control in combination with other expected favorable effects was the main reason for initiation of SGLT2I in *n* = 19 patients. SGLT2I prescribed was dapagliflozin 10 mg in *n* = 20 patients and 5 mg in *n* = 2 patients. Empagliflozin, which was not approved for CKD at the time of the study, was not used in this cohort. N = 10 patients received medication with loop diuretics at baseline (*n* = 9 torasemide, *n* = 1 furosemide).

**TABLE 1:** Characteristics of the study cohort.

	*n*	Median (quartiles) or number
Age, years	22	61 (54; 65)
Sex	22	male 16/female 6
Reason for initiation of SGLT2I	22	*n* = 17 therapy of DM + other favorable effects
		*n* = 2 IgAN recurrence (*n* = 1 also with DM)
		*n* = 1 PVAN
		*n* = 2 elevated HbA1c without manifest DM + other favorable effects
Dose of dapagliflozin	22	5 mg 2/10 mg 20
eGFR (MDRD), mL/min/1.73m^2^	22	38.6 (33.0–57.5)
Albuminuria, mg/g Crea	21	50 (22; 145)
OH, l/1.73m^2^	21	1.3 (0.3; 2.5)
Diuretic therapy	22	No diuretic therapy *n* = 10
		Furosemide *n* = 1
		Torasemide *n* = 9
		Hydrochlorothiazide (HCT) *n* = 2
RAAS inhibitor	22	Ramipril *n* = 7, Enalapril *n* = 1, Candesartan *n* = 10, no *n* = 4
Immunosuppressive therapy	22	Tacrolimus/MMF/Prednisolone 5 mg *n* = 11
		Tacrolimus/MMF *n* = 2
		CSA/MMF/Prednisolone 5 mg *n* = 3
		CSA/MMF *n* = 2
		Sirolimus/MMF/Prednisolone 5 mg *n* = 1
		Sirolimus/Prednisolone 5 mg *n* = 1
		Tacrolimus/Everolimus/Prednisolone 5 mg *n* = 1
		Tacrolimus/Azathioprine *n* = 1
Patients with DM	22	*n* = 18 (pre-existing *n* = 10, PTDM *n* = 8)
HbA1c, %	20	6.6 (6.1; 7.6)

Abbreviations: DM, diabetes mellitus; SGLT2I, SGLT2 inhibitor; GFR, glomerular filtration rate (estimated by CKD-EPI, formula); PVAN, polyoma virus associated nephropathy; OH, overhydration measured by bioimpedance spectroscopy; MMF, mycophenolate mofetil; CSA, cyclosporine A; PTDM, post-transplantation diabetes mellitus.

### Allograft Function

Median estimated GFR (eGFR, estimated with MDRD formula) at BL was 39 (IQR 33–58) mL/min/1.73 m^2^ (Table 1) and decreased by −2 (IQR −10 – 1) mL/min/1.73 m^2^ directly after initiation of SGLT2I without further decrease during 6 months of FU (Table 2). Median albuminuria at BL was 33 (75–174) mg/g creatinine and tended to decrease but without significant changes during FU (Table 2).

**TABLE 2:** Course of parameters during FU.

	BL value	FU 1 (1 week)	FU 2 (1 month)	FU 3 (3 months)	FU 4 (6 months)	FT
Actual FU time, days	0	7 (6; 11)	39 (33; 47)	96 (82; 107)	187 (174; 272)
Number of patients	22	20	16	20	19
**Delta values to BL**					
GFR (MDRD), mL/min/1.73 m^2^	40 (34; 58)	−2 (-10; 1)*	−4 (−5; 4)	**−4** (**-10; 1**)*	−4 (−7; 0)	n.s.
Albuminuria, mg/g Crea	75 (33; 174)	−6 (−31; 19)	−1 (−21; 18)	−12 (−86; 18)	−9 (−39; 9)	n.s.
HbA1c, %	6.1 (6.6; 7.6)	−0.1 (−0.2; 0.0)	−0.1 (−0.4; 0.1)	0.0; (−0.5; 0.4)	0.0 (−0.5; 0.2)	n.s.
Fasting plasma glucose, mg/dL	92 (112; 135)	−7 (−18; 5)	−2 (−19; 6)	−1 (−21; 12)	4 (−8; 14)	n.s.
Body weight, kg	85.2 (75.6; 90.6)	−0.3 (0.8; 0.5)	−0.8 (−1.5; 1.1)	−0.6 (−3.1; 1.4)	−2.3 (−4.4; 1.0)	n.s.
BMI, kg/m^2^	27.3 (25.7; 30.4)	−0.1 (0.3; 0.2)	−0.4 (−0.5; 0.4)	−0.3 (−0.9; 0.5)	−0.9 (−1.8; 0.4)	n.s.
ATM, kg	41.7 (28.9; 48.2)	0.5 (−1.2; 3.4)	−0.6 (−2.3; 1.2)	0.5 (−1.9; 2.5)	1.8 (−4.2; 4.2)	n.s.
FTI, kg/m^2^	13.9 (8.9; 17.1)	0.2 (−0.4; 1.1)	0.1 (−0.6; 0.7)	0.2 (0.7; 1.1)	0.5 (−1.3; 1.4)	n.s.
LTM, kg	39.9 (32.3; 44.3)	−0.25 (−4.0; 1.4)	0.4 (−0.8; 1.4)	−0.9 (−3.3; 1.0)	−1.2 (−5.3; 0)	n.s.
LTI, kg/m^2^	13.6 (11.1; 14.8)	−0.1 (−1.3; 0.6)	0.1 (−0.3; 0.3)	−0.4 (−1.1; 0.3)	−0.3 (−1.7; 0.1)	n.s.
OH, l/1.73 m^2^	1.3 (0.3; 2.5)	−0.4 (−0.8; 0.1)	0.2 (−0.7; 0.4)	−0.3 (−1.0; 0.4)	0.1 (−0.8; 0.3)	n.s.
ECW, l/1.73 m^2^	16.6 (15.4; 17.5)	−0.3 (-0.7; 0.0)*	−0.2 (−0.5; 0.6	−0.5 (−1.0; 0.3)	−0.5 (−0.7; 0.2)	n.s.
Plasma renin, ng/L	21 (5; 57)	0 (0; 5)	0 (0; 4)	0 (−5; 5)	3 (0; 12)	n.s.
Plasma aldosterone, ng/L	156 (132; 211)	45 (5; 114)*	19 (-2; 85)*	35 (−7; 69)	49 (6; 93)*	n.s.
**Absolute values**						
Glucosuria, g/g crea		10 (3; 24)	5 (3; 22)	14 (3; 23)	11 (6; 25)	n.s.

Values are Median and interquartile range. *p < 0.05 with *p*-values from Wilcoxon test to baseline with Bonferroni correction for multiple testing (printed bold if significant). Friedman test (FT) was performed to test for significant changes during total FU period.

Abbreviations: BL, baseline; FU, follow up; FT, friedman test; OH, overhydration measured by bioimpedance spectroscopy; ECW, extracellular water measured by bioimpedance spectroscopy; GFR, glomerular filtration rate; BMI, body mass index; ATM, adipose tissue mass; FTI, fat tissue index; LTM, lean tissue mass; LTI, lean tissue index.

### Glucose Metabolism and Body Composition

**FIGURE 2:**
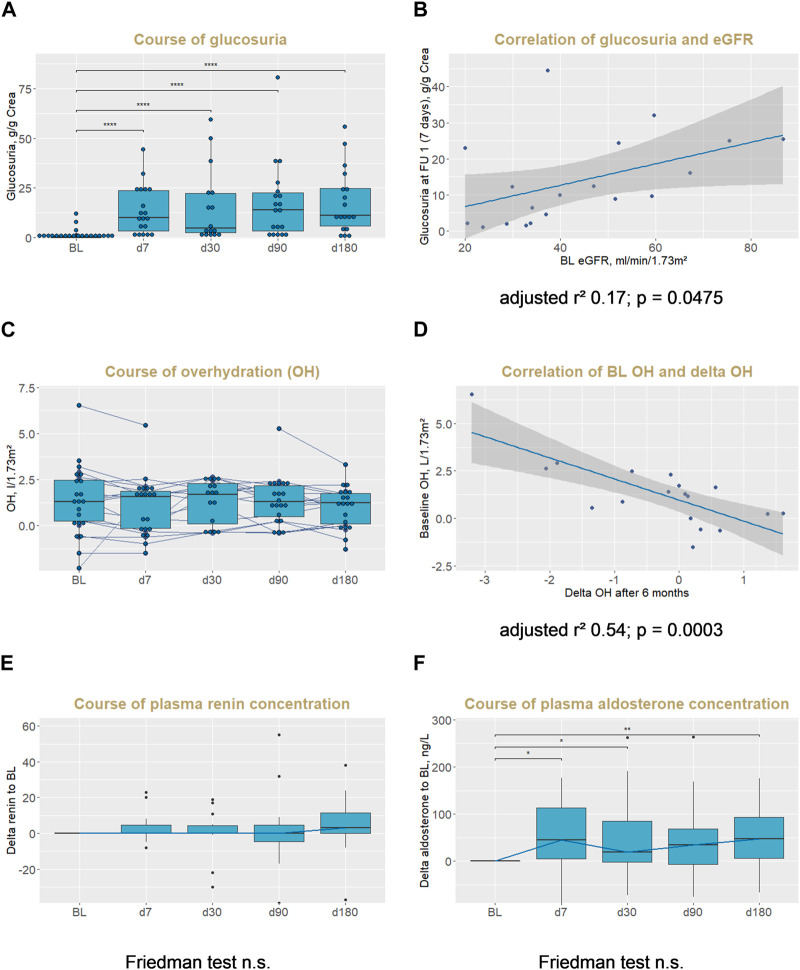
Glucosuria, overhydration, plasma renin and aldosterone concentration after initiation of SGLT2 inhibitors in KTRs. Course of glucosuria [**(A)**, absolute values] and correlation of glucosuria with eGFR **(B)**; course of overhydration [**(C)**, absolute values] and correlation of baseline overhydration with delta overhydration to baseline after 6 months **(D)**; and course of plasma renin [**(E)**, delta values to baseline] and aldosterone [**(F)**, delta values to baseline] concentration. *p* values in **(A,E,F)** are from Wilcoxon tests, with bonferroni correction for multiple testing: *p < 0.05, **p < 0.01, ***p < 0.001, ****p < 0.0001. Note that the distances of x-axis in **(A,C,E,F)** are not representative of follow up time intervals. Abbreviations: KTRs, kidney transplant recipients; BL, baseline; FU, follow up; eGFR, estimated glomerular filtration rate with MDRD formula; OH, overhydration.

Glucosuria was present from the first FU visit 1 week after initiation of the SGLT2 inhibitor with a median urinary glucose concentration at FU 1 of 10 (3–24) g/g creatinine (Table 1). Glucosuria remained stable during further FU (Figure 2A). The degree of urinary glucose concentration correlated with eGFR and was lower in patients with lower eGFR (Figure 2B). There was no significant change of HbA1c or fasting plasma glucose during FU (Table 2).

Median BL BMI was overweight with 27.3 (25.7–30.4) kg/m^2^. Although BMI and body weight tended to decrease during 6 months FU, the reductions were not significant (Table 2). There was no significant change of adipose tissue mass (ATM), fat tissue index (FTI), lean tissue mass (LTM) or lean tissue index (LTI) during 6 months FU (Table 2).

### Overhydration (OH) and Plasma Renin and Aldosterone Concentration

There was a wide range of OH at BL (Figure 2C BL) with a median OH of 1.3 (0.3–2.5) L/1.73 m^2^. During follow-up, range of OH was visually narrowed down and fewer patients had values of OH above 2.0 L/1.73 m^2^ (Figure 2C). This was reflected by a significant correlation of BL OH and delta OH after 6 months, where patients with higher BL OH had a greater decrease of OH (adjusted r^2^ = 0.54, *p* = 0.0003, Figure 2D). Delta OH after 6 months did not correlate with eGFR or delta eGFR, albuminuria or delta albuminuria, or glucosuria. Extracellular water (ECW) decreased in parallel to OH (Table 2).

Loop diuretic therapy was increased in *n* = 2 patients, remained unchanged in *n* = 6 patients and were terminated in *n* = 2 patients by the end of FU (Supplementary Figure S1).

Plasma renin concentration did not change significantly after initiation of SGLT2I or during FU (Table 2; Figure 2E). Plasma aldosterone concentration increased from BL to the first FU visit after 7 days, but there were no further significant changes during 6 months FU (Table 2; Figure 2F).

### Urogenital Infections

Urinary tract infection occurred in *n* = 2 male patients. In the first case, the patient had also had urinary tract infections prior to medication with an SGLT2I, and during complete FU of 6 months, there was only one episode of a lower urinary tract infection with *Klebsiella pneumoniae* found in urinary culture and successfully treated with amoxicillin/clavulanic acid. In the second case, the patient was treated in hospital due to febrile infection and poor glucose control; there was also found *Klebsiella pneumoniae* in urinary culture and infection rapidly subsided with antibiotic therapy with amoxicillin/clavulanic acid; this patient did not wish to continue treatment with dapagliflozin due to an itchy on the forehead with timely but uncertain causal relationship to dapagliflozin.

## Discussion

Our study shows that SGLT2I lead to a correction of fluid overload in those patients with elevated overhydration at baseline, while in euvolemic KTRs fluid status remained stable without fluid loss or reduction of body water below the reference range. These results are in line with previous findings in non-transplant CKD patients, where SGLT2I also lead to a reduction in patients with fluid overload without causing dehydration [7, 8]. In KTRs, there is one smaller previous study also using bioimpedance analysis to investigate volume homeostasis after initiation of SGLT2I, that reported a reduction of fluid overload after 4 weeks (*n* = 14); in this study, mean overhydration returned to initial value after 12 months, but with a decreased sample size of *n* = 8 and large standard deviation, making interpretation uncertain [11]. As there is persistent glucosuria under therapy with SGLT2I, we should actually expect a lasting osmotic effect, but there was no ongoing fluid loss after SGLT2 inhibition in all these cohorts. Therefore, counter-regulation mechanisms promoting stabilization of extracellular water seem to be active. One potential mechanism is activation of renin angiotensin aldosterone system (RAAS). In normally hydrated patients with diabetes mellitus and normal kidney function, we had observed a transient loss of extracellular water, which was counter-regulated by RAAS activation [4]. In non-transplant CKD cohorts, decrease of fluid overload was accompanied by a tendential but not significant increase of renin and aldosterone [7, 8]. In our present cohort of KTRs, SGLT2I did not lead to an increase in renin and elicited only a moderate response in aldosterone concentrations, indicating that denervation of the kidney during transplantation might have an impact on the RAAS activation. Another potential mechanism promoting stabilization of fluid status with SGLT2I is water conservation by the antidiuretic hormone (ADH/Vasopressin). The vasopressin surrogate marker copeptin has been shown to increase after SGLT2 inhibition in non-transplant CKD patients [8]. Marton and colleagues proposed an effect called aestivation, that is known as an evolutional survival strategy in energy and water shortage, to become active with SGLT2 inhibition [25]. Aestivation-like changes of metabolism with nitrogen transfer for production of organic osmolytes with parallel water conservation via ADH might prevent the glucose-driven osmotic diuretic effect of SGLT2I and contribute to renoprotective effects of SGLT2I [25]. Most recently, this effect was examined in patients with heart failure, where, in accordance with this finding, SGLT2 inhibition lead to increased serum copeptin levels and decreased free water clearance [26].

The denervation of the transplanted kidney might also lead to a different reaction to changes in volume status than the native kidneys. Our current study shows, however, that correction of fluid overload without ongoing fluid loss with SGLT2I is also present in the cohort of KTRs, suggesting that this effect of SGLT2I is independent from innervation of the kidneys. Our observations overall confirm the safety of SGLT2I with respect to the risk of dehydration after kidney transplantation and emphasize a potential benefit of SGLT2I, particularly in patients with sub-clinic or obvious fluid overload. Of note, we investigated the use of SGLT2I in stable KTRs at least 6 months after the kidney transplantation, and so the impact on fluid status may be different early after kidney transplantation. Prospective outcome studies investigating the reno-protective effects of SGLT2I in KTRs are still pending. The Renal Lifecycle Study (NCT05374291) investigating effects of dapagliflozin on renal and cardiovascular outcomes includes KTRs and is currently recruiting.

Our study confirms that glucosuria with SGLT2I depends on the kidney function and is reduced in lower GFR after kidney transplantation. This has already been demonstrated in KTRs [19] and is in line with previous findings in non-transplant patients, where urinary glucose excretion also decreased with kidney impairment [27]. Due to this dependency of SGLT2I-mediated glucosuria from GFR, we assume that the effect of SGLT2I on blood glucose control is lower in patients with impaired kidney transplant function. This should be borne in mind when selecting a glucose-lowering therapy in these patients. Together with the limited study size, this probably explains why we did not observe significant changes of fasting plasma glucose or HbA1c in our cohort.

Although body weight tended to decrease in a range reported earlier [11–14, 16–18] there was no reduction of adipose tissue mass. Likewise, adipose tissue was not reduced by SGLT2I in a previous smaller cohort of KTRs [11]. This might be on account of lower calorie losses in the urine due to decreased eGFR. Furthermore, our cohort was overweight and therefore differed from obese cohorts, the latter showing a greater reduction of body weight and decrease in adipose tissue following initiation of SGLT2 inhibitors [4, 28]. Lean tissue was also not decreased in our cohort, which speaks against loss of muscle mass under therapy with SGLT2I in KTRs. However, effects of SGLT2I on body fat and lean tissue might become more pronounced after longer FU time and might be overseen in our study due to the small cohort size. Especially as there seem to be present, as discussed above, counter regulating effects of ongoing fluid loss like aestivation-like metabolic changes which include consumption of amino acids from muscle tissue, further findings on the course of muscle tissue under SGLT2I must be awaited.

Our study is restricted by the small sample size and limited follow-up period. However, we present new and robust data on the impact of SGLT2I on fluid status and glucosuria after kidney transplantation. Since changes of fluid status are expected soon after the initiation of SGLT2 inhibitors, we monitored respective parameters closely at an early follow up visit after 7 days. We used bioimpedance spectroscopy as a reliable and investigator independent tool for intra-individual change of fluid status over time, producing clinically applicable parameters. Our findings promote the safety of SGLT2 inhibitors following kidney transplantation and support a broader use that will lead to further clinical experience.

In conclusion, our study demonstrates a correction of fluid overload after initiation of SGLT2I without risk of volume depletion and promotes the safety of SGLT2I therapy in patients after kidney transplantation. Glucosuria, together with effects of SGLT2I on blood glucose control and body weight, is reduced in lower kidney allograft function.

## Data Availability

The raw data supporting the conclusion of this article will be made available by the authors, without undue reservation.

## Abbreviations

ATM, adipose tissue mass; BCM, Body Composition Monitor (Fresenius Medical Care); BL, baseline; BMI, body mass index; CKD, chronic kidney disease; ECW, extracellular water; eGFR, estimated glomerular filtration rate, estimated with MDRD formula; FTI, fat tissue index; FU, follow up; ICW, intracellular water; KTRs, kidney transplant recipients; LTI, lean tissue index; LTM, lean tissue mass; OH, overhydration; PTDM, post-transplantation diabetes mellitus; RAAS, renin angiotensin aldosterone system; SGLT2I, SGLT2 inhibitors; TBW, total body water.
