# Supplementary material for: SGLT2 Inhibitors Correct Fluid Overload in Adult Kidney Transplant Recipients—A Prospective Observational Study
Source: Transpl Int. 2024 Jun 10;37:12879. doi: 10.3389/ti.2024.12879 (PMC11194332; doi:10.3389/ti.2024.12879)
Supplement: Supplementary file 1 [file DataSheet1.PDF]

Suppl. figure 1: Patients with loop diuretic therapy and change of loop diuretic therapy during follow up

Patient	OH at BL	BL loop diuretic therapy	Dose adjustment of loop diuretic therapy at					OH at end of available FU
			BL	FU1	FU2	FU3	FU4	
1	6.2 l/1.73m²	Torasemide 10 mg	0 mg	0 mg	n.a.	10 mg	10 mg	3.3 l/1.73m²
2	0.9 l/1.73m²	Torasemide 10 mg	10 mg	10 mg	20 mg	10 mg	10 mg	0.0 l/1.73m²
3	2.0 l/1.73m²	Torasemide 10 mg	0 mg	0 mg	0 mg	0 mg	0 mg	1.0 l/1.73m²
4	1.3 l/1.73m²	Torasemide 10 mg	10 mg	10 mg	20 mg	20 mg	20 mg	1.4 l/1.73m²
5	1.7 l/1.73m²	Torasemide 10 mg	10 mg	10 mg	10 mg	20 mg	20 mg	1.7 l/1.73m²
6	1.6 l/1.73m²	Torasemide 10 mg	10 mg	10 mg	15 mg	15 mg	10 mg	2.2 l/1.73m²
7	2.6 l/1.73m²	Torasemide 20 mg	20 mg	20 mg	n.a.	20 mg	20 mg	0.6 l/1.73m²
8	-2.3 l/1.73m²	Torasemide 50 mg	50 mg	50 mg	n.a.	n.a.	n.a.	1.8 l/1.73m²
9	0.0 l/1.73m²	Furosemide 20 mg	0 mg	n.a.	0 mg	0 mg	0 mg	0.2 l/1.73m²
10	3.5 l/1.73m²	Torasemide 10 mg	10 mg	n.a.	10 mg	10 mg	n.a.	1.2 l/1.73m²

Different shades of grey represent dose of loop diuretic.

Abbreviations: BL, baseline; FU, follow up; n.a. = not available; OH, overhydration.
